# Improving the Reprocessing Quality of Flexible Thermolabile Endoscopes: How to Learn from Mistakes

**DOI:** 10.3390/ijerph18052482

**Published:** 2021-03-03

**Authors:** Beatrice Casini, Benedetta Tuvo, Emanuele Marciano, Giuliana Del Magro, Giulia Gemignani, Grazia Luchini, Maria Luisa Cristina, Anna Laura Costa, Guglielmo Arzilli, Michele Totaro, Angelo Baggiani, Gaetano Privitera

**Affiliations:** 1 Department of Translational Research, New Technologies in Medicine and Surgery, University of Pisa, 56126 Pisa, Italy; b.tuvo@studenti.unipi.it (B.T.); anna.costa@med.unipi.it (A.L.C.); g.arzilli3@studenti.unipi.it (G.A.); michele.totaro.unipi@hotmail.com (M.T.); angelo.baggiani@med.unipi.it (A.B.); gaetano.privitera@med.unipi.it (G.P.); 2 Endoscopy Service Division, University Hospital of Pisa, 56126 Pisa, Italy; e.marciano@ao-pisa.toscana.it (E.M.); g.delmagro@ao-pisa.toscana.it (G.D.M.); 3 Medical Direction, University Hospital of Pisa, 56126 Pisa, Italy; g.gemignani@ao-pisa.toscana.it (G.G.); grazia.luchini@ao-pisa.toscana.it (G.L.); 4 Department of Health Sciences, University of Genova, 16132 Genova, Italy; maria.luisa.cristina@galliera.it

**Keywords:** endoscope reprocessing, duodenoscope, microbiological surveillance, *KPC*, NDM, *Klebsiella pneumoniae*

## Abstract

**Background:** Failure in the reprocessing of thermolabile flexible endoscopes has been reported as one of the most important threats to patient health. **Method:** A case report and observational study was conducted, from August 2014 to December 2019, in the Digestive Endoscopy Unit of a University Hospital in Italy, where two cases of Klebsiella pneumoniae carbapenemase (*KPC*)-producing *Klebsiella pneumoniae* infections in patients undergoing endoscopic retrograde cholangio-pancreatography were observed. Following the risk/safety management practices, an epidemiological investigation was started, duodenoscopes were removed from use and the reprocessing practices reviewed. Moreover, microbiological surveillance of endoscopes was carried out according to the CDC guidelines. **Results:** In the first phase of sampling, 10/10 (100%) endoscopes were found to be non-compliant, of which 7 showed results for high-concern organisms (HCOs), such as *KPC*-*K. pneumoniae, P. aeruginosa* and *E. coli*. After implementing corrective actions, 12 out of 17 endoscopes were found to be non-compliant (70.5%), of which 8 showed results for HCOs, such as *KPC-K. oxytoca* and *P. aeruginosa*. During the last year of regular microbiological surveillance, only 23% of endoscopes (35/152) were found to be non-compliant, of which 7 showed results for HCOs, such as *NDM-K. pneumoniae, P. aeruginosa* and *A. baumannii*. The crucial issues were related to samples collected from the internal channels of duodenoscopes. **Conclusion:** Managing the risk associated with the reprocessing of digestive endoscopes, through risk assessment at every stage of the process, is important for the prevention of infections associated with the use of these device.

## 1. Introduction

Failure in thermolabile flexible endoscopes reprocessing has been reported as one of the ten most important threats to patient health [1] and is a frequent occurrence due to the complexity and multiplicity of the steps involved in this process. These phases have been partly automated in order to avoid operator-dependent errors; however, it still is important to identify and, consequently, monitor the possible causes of failure in the manual phases of the process. Deviation from the manufacturer’s recommendations can potentially lead to residual microorganisms being left on the device, thus increasing the risk of infection. In this context, microbiological surveillance is a valid tool for detecting procedural errors or microlesions of the endoscope, which can facilitate biofilms formation and cause positivity to microbial cultures [2]. However, infections associated with contaminated endoscopes on which the reprocessing procedure was correctly performed have been reported [3,4,5,6]. Recently, a meta-analytical study estimated a contamination rate of patient-ready duodenoscopes for endoscopic retrograde cholangio-pancreatography (ERCP) of 16.14% ± 0.019 (95% Cl: 12.43–19.85%) after high-level disinfection [7].

In 2015, the Food and Drug Administration required that each of the three manufacturers who produce the duodenoscopes sold in the U.S. (Fujifilm Medical Systems USA, Inc; Olympus Medical Systems Corporation; Pentax of America) conduct postmarket surveillance studies, in order to better understand how duodenoscopes are reprocessed in real-world settings and evaluate the percentage of duodenoscopes that remain contaminated with viable microorganisms after reprocessing according to manufacturer’s instructions. Despite the decline in the number of medical device reports (MDRs) associated with patient infections, observed following the implementation of safety measures to improve reprocessing techniques, the FDA received 205 MDRs from October 2018 to March 2019, a 51% increase over the previous three years. The presence of high-concern organisms (microorganisms more often associated with disease) was demonstrated in more than 5.4% of duodenoscopes, a higher-than-expected frequency of contamination (a 3% increase from the previous 2015 report) [8].

Although contaminated endoscopes have been involved in more health care-associated infections than any other reusable medical device [9], the number of infections still remains underestimated due to factors such as incomplete surveillance, underreporting, asymptomatic infections and infections with prolonged latency.

Until now, infections due to multidrug-resistant microorganisms have been easily recognized and frequently reported [10], but these infections are only a tiny tip of the iceberg [11]. One good estimate of the risk of transmission of infections related to endoscopes is certainly the evaluation of their contamination. As reported in the ESGE/ESGENA Position Statement [12], microbiological surveillance is important for the quality assurance of each phase of the reprocessing, providing information about any deviations, errors and failures of the procedure. Particular attention should be paid to the most crucial devices, such as duodenoscopes and echoendoscopes [13].

In this study we report the experience of a large Digestive Endoscopy Unit in an Italian University Hospital in improving the reprocessing quality of flexible thermolabile endoscopes following the occurrence of Klebsiella pneumoniae carbapenemase (*KPC*)-producing *Klebsiella pneumoniae* infections after ERCP.

## 2. Material and Methods

### 2.1. Setting

This case report and observational study was conducted in a large Digestive Endoscopy Unit (DEU) of an Italian University Hospital in order to assess the quality of endoscope reprocessing activities and the role of these devices in the transmission of patient infections. In 2019, 13,222 colonoscopies, 14,735 gastroscopies and 1100 endoscopic ERCPs and echoendoscopies were performed overall.

In this setting, in August 2014, *KPC*-*K. pneumoniae* bacteremia was observed in two patients who had previously undergone ERCP during hospitalization. According to the risk/safety management practices, an epidemiological investigation was started to verify the role of the duodenoscopes in the infection’s transmission. Duodenoscopes were removed from use and the reprocessing practices were reviewed, while microbiological surveillance of the endoscopes was carried out to assess the efficacy of the reprocessing. This surveillance only later became systematic, with the definition of a monitoring program providing for the sampling of crucial endoscopes (duodenoscopes and echoendoscopes) once a month and of the other instruments (gastroscopes and colonoscopes) every three months.

*KPC*-*K. pneumoniae* strains isolated from patients by the hospital-based microbiology laboratory and from the endoscopes during the microbiological surveillance were collected and genotyped. All isolates were investigated for *KPC* production based on phenotypic inhibitory activity with phenylboronic acid [14]. KPC subtyping was performed using PCR and sequencing of blaKPC genes [15]. Isolates were typed by PFGE of XbaI-digested genomic DNA using the Chef-DRw III System (Bio-Rad, Marnes-La-Coquette, France). Electrophoresis was run at 14 °C with a pulse time of 5–60 s at 6 V/cm on a 120° angle in 0.5× Tris/borate/EDTA (TBE) buffer for 22 h. Multilocus Sequence Typing (MLST) was performed as previously reported [16].

### 2.2. Reprocessing Procedure

Reprocessing of the gastrointestinal endoscopes was carried out in accordance with the manufacturer’s recommendations and indications proposed by guidelines [17,18,19,20,21]. The process involved the following phases: bedside pre-cleaning and then, immediately afterward, in a separate purpose-designed reprocessing room, a leak test, manual cleaning, high-level disinfection in an automatic flexible endoscope reprocessor and, after a visual inspection, forced air drying before storage in drying/storage cabinets or an unventilated vertical cabinet. The number of drying/storage cabinets available in the DEU was not sufficient to house all the endoscopes.

The audit visits carried out to assess the quality of the reprocessing procedure were conducted using the ARS Tuscany checklist as a reference [22].

### 2.3. Microbiological Surveillance

**Figure 1:**
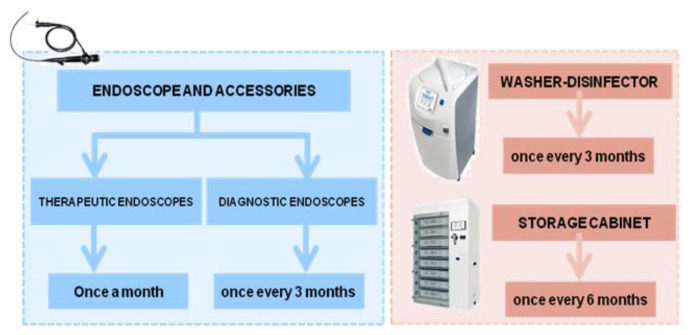
Microbiological surveillance program.

After the first cases of infection that were probably related to ERCP, microbiological surveillance was carried out, according to the method proposed by the ESGE–ESGENA guideline [20], on selected endoscopes in order to ascertain the reprocessing efficacy. Starting from August 2015, the monitoring protocol was modified to use the new technique proposed by the CDC guidelines [23,24]. Both methods required the sampling of each channel and the outer surfaces of endoscopes, the final rinse water of washer disinfectors, the supply water and the water contained in the irrigation bottle, in addition to the surfaces of the storage cabinets (Figure 1).

#### 2.3.1. Endoscope

Following the ESGE–ESGENA guideline [20], microbiological testing was carried out separately for each channel and outer surface. Briefly, under aseptic conditions, sterile swabs moistened with 0.9% sterile saline were taken from the distal end, the valve ports and the bridge elevator (in duodenoscopes and echoendoscopes); moreover, all channels were flushed with 20 mL of sterile saline (5 mL in the elevator channel because of its small lumen). Each swab was extracted in 10 mL of Tryptic Soy Broth (TSB, Merck Millipore, Burlington, MA, USA) using a vortex. A portion of each liquid sample was analyzed for total viable count (TVC) and specific microorganism detection (Enterobacteriaceae, *Pseudomonas aeruginosa* and staphylococci). In addition, antimicrobial susceptibility tests were performed by VITEK 2 (Biomerieux, Marcy-l’Étoile, Francia).

The new protocol introduced in August 2015—the “flush-brush-flush” technique, echoing the recommendations of the CDC guideline [23,24]—was used for the sampling of each instrument channel and the elevator recess of duodenoscopes, with the exception of the elution solution. A 0.01 M Phosphate Buffered Saline solution (PBS, Merck KGaA, Darmastadt, German) with 0.02% of Tween 80 (Merck KGaA, Darmastadt, German) was used instead of sterile ionized water because it was considered more suitable for recovery of microorganisms’ vitality [25]. A final volume of about 45 mL was obtained from all the channels sampled simultaneously and collected in a container in which the same volume of Dey Engley neutralizing broth (DE broth, Sigma Aldrich, Milan, Italy) was added in order to neutralize any trace of chemicals that might have limited detection of microorganisms. The PBS–Tween 80 solution was used to moisten a sterile swab that was taken from the distal end and cumulated to the liquid sample.

The entire volume of the sample was filtered through a 0.45 μm filter and the membrane was placed on a plate of Blood Agar (VWR International PBI, Radnor, PA, USA) and incubated at 35–37 °C for 72 h. Colony-forming units (CFUs) were enumerated and each species identified to distinguish high-concern organisms from low/moderate-concern organisms.

Starting from late 2018 and until the end of 2019, microbiological surveillance was extended to all the endoscopes, on a monthly basis for duodenoscopes/echoendoscopes and quarterly for the others (gastroscopes and colonoscopes). For the latter, one endoscope was tested at each sampling session in rotation, ensuring that each one was sampled at least once at the end of the year.

#### 2.3.2. Water of the Irrigation Bottle

Samples were taken at the end of the endoscopic sessions of the day and the TVC was assessed in 1 mL according to the method proposed in the standard ISO 6222:1999.

#### 2.3.3. Endoscope Washer-Disinfector

In order to evaluate the quality of the water used by the ISO 15883-4-compliant washer disinfector, samples were taken at the inlets of the machines and at the final rinses. The sampling was carried out every three months according to the Italian law (D. Lgs. 31/2001) for the inlet water and according to ISO 15883-4 for the final rinse water. Samples of 2 L for each type of water were collected in sterile bottles containing sodium thiosulphate to neutralize any residual disinfectant.

In the inlet water the absence, in 250 mL, of *P. aeruginosa* was ascertained according to ISO 16266:2008; in 100 mL, the absence of coliform bacteria, and in particular *E. coli*, was ascertained according to 9308-1:2014/Amd1:2016, while that of enterococci was ascertained according to ISO 7899-2:2003. The measurement of TVC was performed by inclusion in Plate Count Agar (Oxoid, UK), following ISO 6222:1999, and the acceptable limits of ≤100 CFU/mL and ≤10 CFU/mL, respectively at 22 °C and 36 °C, were applied.

In the final rinse water, the absence, in samples of 100 mL, of *P. aeruginosa* was ascertained according to ISO 16266:2008; the absence of atypical mycobacteria was ascertained according to the protocol reported by Casini et al. [26]; and that of *Legionella* spp. was ascertained according to ISO11731:2017. Measurements of TVC at 22 °C and 36 °C were evaluated according to ISO 6222:1999 (acceptable limit: ≤1 CFU/100 mL).

#### 2.3.4. Storage Cabinet

Surfaces of unventilated vertical cabinets and of standard EN 16442:2015-compliant storage cabinets (5 and 4, respectively) were sampled every six months to verify the adherence to the microbiological limit of ≤25 CFU/24 cm^2^. Following ISO 14698-1, Rodac plates with diameters of 55 mm, containing Plate Count Agar (PCA) with neutralizers (VWR International PBI, Radnor, PA, USA), were used for TVC enumeration. For each tray, four contact plates were used: at the two diagonally opposite corners, one in the center of the tray and one for the lid (on the internal surface). Contact plates were incubated aerobically at 36 °C for 48 h. The same method was applied in the sampling of the surfaces inside the unventilated vertical cabinets.

## 3. Results

Following the occurrence of post-ERCP *KPC*-*K. pneumoniae* infections, in August 2014 patients were followed up and clinical data collected. In this hospital, a systematic surveillance for multi-drug-resistant organism colonization was in place through weekly rectal swabs and/or bronchial aspirate sampling in patients admitted in the more critical areas (ICUs, transplant surgery, oncology, etc.). Following the *KPC*-*K. pneumoniae* infections, the hospital’s review board established that this investigation was a quality assurance practice and therefore rectal swabs were also required for patients undergoing endoscopy with deep sedation. 

### 3.1. Cases Report

The first patient (patient A), a female aged 69 with an admission diagnosis of pancreatic cancer, had ERCP during the first day of hospitalization, had an esophagogastroduodenoscopy (EGD) nine days afterwards and on the eleventh day she had a duodenocephalopancreasectomy. After the surgery, she was admitted for three days into the intensive care unit (ICU). Eleven days after ERCP, microbiological analysis evidenced *KPC*-*K. pneumoniae* biliary colonization and, 19 days after ERCP, a bloodstream infection by *KPC*-*K. pneumoniae* was diagnosed. Two days later, the presence of *KPC*-*K. pneumoniae* was also found in the drainage fluid of the surgical wound and in the rectal swab. A week after modifying the therapy, the patient was apyretic and blood cultures were negative; on the 15th day, she was discharged with a negative rectal swab. The second patient (patient B), a female aged 69 with an admission diagnosis of gallbladder cancer, had ERCP on the eighth day after hospitalization and by the eleventh day she was pyretic because of an extended spectrum β lactamase (ESBL)-producing *E. coli* bloodstream infection. After 22 days of antibiotic therapy, the patient was again pyretic for a *KPC*-*K. pneumoniae* bloodstream infection. *KPC*-*K. pneumoniae* colonization was found with rectal swab and biliary excretion analysis. The patient was discharged with colonization and after nine months had a readmission for *KPC*-*K. pneumoniae* sepsis.

Molecular typing revealed that all the *K. pneumoniae* strains were *KPC*-producing, showing the *KPC*-3 variant and the ST512 by MLST analysis. PFGE results strongly agreed with the MLST data, showing one major PFGE group (Pt A) belonging to ST512. Strains isolated in patient A from bile, blood, drainage fluid and rectal swab appeared to be clonally related since the same Pulsetype A1 (PtA1) was detected in all the samples. This genotype, ST 512/Pt A1, was also detected in the sample collected from the duodenoscope used on patient A (see below). Three closely related pattern variants (Pt A2, Pt A3 and Pt A4) were identified in samples (blood, rectal swab and biliary excretion) taken from patient B and from the duodenoscope used on her, sharing 87% similarity in PFGE patterns. In the pattern profile, *K. pneumoniae* belonging to ST101 was added as a control strain with a 65% similarity with Pt A profiles.

### 3.2. Risk/Safety Management Plan Results

During the audit visits conducted from August 2014 in the Digestive Endoscopy Unit some crucial issues were highlighted concerning the reprocessing phases.

The enzymatic solution used in the pre-cleaning phase for the decontamination of endoscopes was not discarded after each endoscopic procedure. The cleaning phase was not conducted separately from the high-disinfection phase and there were no procedures for the clean–dirty path of the instruments. The dimensions of the washbasin for the manual cleaning were not adequate to permit the complete immersion of the endoscope and brushes were used several times without being disinfected. Moreover, a source of forced air for the drying phase was not present and endoscopes were stored in an unventilated cabinet when still damp. A procedure for the disinfection of the internal surfaces of the cabinets was not present. A traceability system was in place to allow patients to be recalled by entering the report issued by the high-level disinfection automatic reconditioner into the patient’s medical record, but no traceability was in place to verify the storage time in the cabin and, for this reason, some endoscopes were stored for more than three days.

### 3.3. Corrective Actions Implemented after the Cases of Infection

Following the identification of crucial issues associated with the reprocessing procedure, improvement actions were envisaged that affected both structural and operational aspects. These actions were subsequently modified in light of the latest Position Statement published by ESGE–ESGENA [12].

#### 3.3.1. Activation of Active Screening

Starting from September 2014, for *KPC*-*K. pneumoniae* and other alert organisms’ colonization statuses, active screening by rectal swab was undertaken in patients undergoing endoscopy with deep sedation. Active surveillance of colonization/infection cases following the execution of ERCP (post-exposure epidemiological investigation) was also introduced. This screening is still active today.

In the endoscope reprocessing procedure, disposable devices were adopted where necessary and feasible.

#### 3.3.2. Pre-Cleaning Phase

Given the high risk of cross-contamination, the enzymatic solution was eliminated after each procedure. When colonization/infection status for *KPC*-*K. pneumoniae* or other MDR microorganisms was known, the last endoscopic session of the day was dedicated to these patients.

#### 3.3.3. Manual Cleaning Phase

In the Digestive Endoscopy Unit a cleaning room separated from the high disinfection area was not available; for this reason, a clean–dirty path was created to avoid cross-contamination. The staff was adequately trained to respect the path. In December 2014, a sink of appropriate size was installed for the full immersion of endoscopes in detergent solution before brushing activities. The detergent solution was not reused and single-use brushes were adopted based on manufacturer instructions, in particular for the cleaning of crucial endoscope components (such as the elevator mechanism of duodenoscopes). In order to improve the irrigation of the internal channels and the process traceability, the use of the automatic peristaltic pump was introduced in April 2019 (SEB 1000 Soluscope, Aubagne, France). Single-use endoscope components, such as valves and distal caps for some duodenoscopes, were introduced to enable full traceability and to prevent cross-infection.

#### 3.3.4. High-Disinfection Phase

Before improvement actions, endoscopes were subjected to high disinfection with 6% hydrogen peroxide in an EN ISO 15883-1- and 15883-4-complying automatic reprocessor (Endoscope Reprocessing Andsterning System, CISA, Italy). Due to non-compliant rinse water analysis results (as shown below), and the finding of contaminated endoscopes despite the improvement of the manual cleaning procedure, the high disinfection was switched to 0.2% peracetic acid in September 2014. To improve the water quality, and therefore the effectiveness of the microfiltration operated by the disinfection automatic reprocessor, a pre-filtration system was installed at the entrance of each machine, consisting of three polypropylene filters arranged in series, with porosities of 20, 5 and 1 μm respectively (Serie Claris, Pall Corporation, PALL). Subsequently, due to the age of these machines and the problems associated with their maintenance, in August 2018 it was decided to replace them with new automatic reprocessors that used peracetic acid and 900 ppm at 40 °C (Serie 4 Soluscope, France).

#### 3.3.5. Storage Phase

After a visual inspection, endoscopes were carefully dried with forced air drying and stored in unventilated vertical cabinet for temporary storage. Subsequently, in order to increase storage hygienic quality and endoscope shelf life after reprocessing, controlled environment storage cabinets (DSC800 Soluscope, France) complying with EN ISO 16442:2015 were installed in September 2018. Strict operational separation of dirty and clean storage areas was ensured to avoid recontamination of reprocessed endoscopes.

#### 3.3.6. Staff Training

Staff was re-trained in the changed reprocessing procedure and in the use of the automatic machines. Moreover, the staff was adequately trained to respect the clean–dirty path to avoid cross-contamination. A formal training program lasting one week was followed by the endoscopy and retreatment staff and the competencies acquired verified by a questionnaire, submitted before and at the end of the course. Increases in knowledge were demonstrated (data not shown) and the experience was since repeated quarterly. The course was organized with a theoretical part and a practical one, where the ability of the staff to effectively perform the manual washing procedures was assessed by ATP detection, used for teaching purposes as a potential marker of cleaning adequacy.

#### 3.3.7. Transport of the Endoscopes

A crucial issue was found in the transport of endoscopes used in operating theaters during night emergencies. The time before reprocessing did not comply with the recommendations of the guidelines (start of the manual cleaning phases within about 30 min from the end of the procedure) [12]; therefore, a dedicated procedure was activated with the hospital logistics sector in order to speed up the delivery of endoscopes to the reprocessing room.

#### 3.3.8. Microbiological Surveillance Protocol

The procedure used in the microbiological surveillance of endoscopes was modified in light of new evidence reporting greater recovery with the flush-brush-flush method. Moreover, a monitoring program was established, providing for the sampling of crucial endoscopes (duodenoscopes and echoendoscopes) once a month and of the other instruments (gastroscopes and colonoscopes) every three months. Any endoscope found to be non-compliant was placed in quarantine until the next negative outcome of the microbiological surveillance.

### 3.4. Microbiological Surveillance

**Figure 2:**
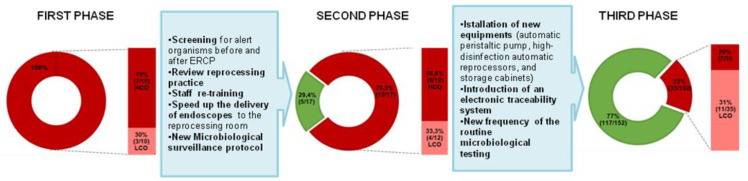
Results obtained during the three phases of the microbiological surveillance. Compliant results in green and non-compliant results in red.

In the first phase of the monitoring, between August and September 2014, ten endoscopes (four colonoscopes, four duodenoscopes and two gastroscopes) were sampled after the 6% hydrogen peroxide high disinfection. All the analyzed endoscopes were found to be non-compliant, being contaminated in one or more sampled sites (Table 1): in seven endoscopes at least one colony of indicator microorganisms was isolated (such as *Escherichia coli*, *Klebsiella pneumoniae* and *Pseudomonas aeruginosa)* and three of these also exceeded the microbial limit recommended by the ESGE/ESGENA (>20 CFU/channel); there were also three endoscopes for which indicators were not isolated (Table 1). Interpreting the data according to the CDC recommendations, the non-conformities were the same: 30% of endoscopes showed >100 CFUs of low/moderate-concern organisms and the remaining 70% showed growth of high-concern organisms (Figure 2).

In two duodenoscopes, *KPC*-producing *Klebsiella pneumoniae* was detected in the liquid sample from the biopsy channel, with the TVC value exceeding the suggested limits at 27 and 350 CFU/20 mL, respectively.

**Figure 3:**
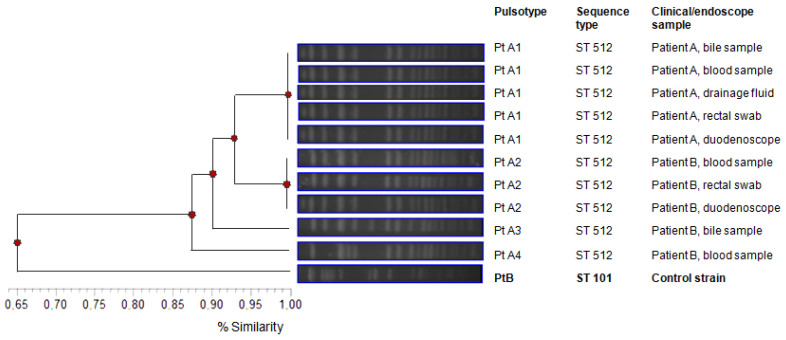
Genotyping of Klebsiella pneumoniae carbapenemase (*KPC*)-*K. pneumoniae* strains isolated from patients and endoscopes.

The genotype of the *Klebsiella pneumoniae* strain isolated from the duodenoscope was the same as that isolated in the patient’s clinical samples (Pt A1/ST 512 and Pt A2-A3-A4/ST512, respectively), confirming the role of duodenoscopes as the cause of infection (Figure 3).

During the first phase of the surveillance, the inlet water of the 6% hydrogen peroxide automatic reprocessor was found to be non-compliant with the Italian law (D. Lgs. 31/2001), with a high TVC at 36 °C, equal to 536 CFU/mL, and detection of *P. aeruginosa*. Non-conformities were also found in the final rinse water (TVC at 36 °C of 390 CFU/mL and *P. aeruginosa* detection).

Following the implementation of corrective actions and the conversion to 0.2% peracetic acid for high disinfection, 17 endoscopes (6 colonoscopes, 10 duodenoscopes and 1 gastroscope) were sampled between August 2015 and May 2016 (second phase). For these tests, the new sampling method proposed in the CDC guidelines was applied and 70.5% (12/17) of endoscopes were found to be non-compliant, 33.3% (4/12) due to high TVCs for low-concern organisms and 66.6% (8/12) due to high-concern organisms, with 4 of these latter also showing high TVCs. The crucial issues were again found in the analysis of the liquid samples collected from the internal channels of duodenoscopes: six out of ten were found to be non-compliant, with three of these showing growths of *Klebsiella pneumoniae*, which is sensitive to the main classes of antibiotics, as the most frequently isolated species, while in one sample *KPC*-producing *Klebsiella oxytoca* was also detected (Figure 2, Table 2).

Following the installation of the pre-filtration system at the entrance of each automatic reprocessor, four machines were sampled and 100% of the inlet (4/4) and final rinse water (4/4) samples were compliant.

In August 2018, the reprocessing room was completely renovated and new automatic machines were installed. In the cleaning phase, to improve the irrigation of the internal channels and ensure full traceability, an automatic peristaltic pump was introduced (SEB 1000 Soluscope, Aubagne, France), as well as five high-disinfection peracetic acid automatic reprocessors (900 ppm at 40 °C) (Serie 4 Soluscope, France) compliant with ISOs 15883-1 and 15883-4. In the storage room, four controlled environment storage cabinets (DSC800 Soluscope, France), complying with EN ISO 16442:2015, were installed. In addition, an electronic traceability system was introduced thanks to the use of the IT software Soluscope (Soluscope, Aubagne, France), which made it possible to track the patient on which the endoscope was used in real time, verify the quality of the reprocessing and thus achieve patient safety.

Starting from January 2017, a plan for microbiological monitoring activities was also designed in order to verify the reprocessing process. In accordance with UNI TR 11662:2016, the frequency of routine testing was established for endoscopes, washer disinfectors, accessories and the water supply used in endoscopy.

In the last year of monitoring, from January 2019 to December 2019 (not considering the year 2020 during which endoscopic activity was strongly reduced due to the COVID-19 pandemic [27]), 152 endoscopes were sampled after reprocessing procedures, of which 72 were duodenoscopes, 24 were echoendoscopes, 32 were gastroscopes and 24 were colonoscopes.

A total of 35 out of 152 (23%) endoscopes were found to be non-compliant. While all the samples collected from the echoendoscopes were found to be compliant (two echoendoscopes sampled 24 times), 15 duodenoscopes were not compliant (15/72, 20.8%) and showed the growth of high-concern organisms, mainly enterobacteria, including *Klebsiella pneumoniae* as well as Pseudomonadaceae (Table 3). In four duodenoscopes, in the liquid collected from the internal channels, the TVCs were found to be higher than 100 CFU/channels (quantity range of 144–300 CFU/channels) and in two cases associated with enterobacteria. Seventeen duodenoscopes were overall considered non-compliant.

In the gastroscopes, high TVCs of low/moderate-concern microorganisms were detected in 4 out of 32 (12.5%) liquid samples and high-concern organisms were isolated in 8 out of 32 (25%) (Table 3). Eleven gastroscopes were overall considered non-compliant. Of note was the isolation of New Delhi metallo-β-lactamase (NDM)-producing *K. pneumoniae* from two gastroscopes, one of which used on a patient undergoing surgery of the esophagus, with intestinal tract colonized by this bacterium. After the theatre session, which took place as a night emergency, the gastroscope was subjected to the pre-cleaning phase, but the reprocessing was only completed the following morning.

Regarding colonoscopes, 4 out of 24 liquid samples from internal channels showed TVCs of low/moderate-concern organisms greater than 100 CFU/channels (quantity range of 300–1043), and high-concern organisms, represented by *E. coli*, *Staphylococcus* spp. and *P. aeruginosa*, were also found. Seven colonoscopes were overall considered non-compliant (Table 3).

All endoscopes found to be non-compliant (35 of 152) were sampled again after the reprocessing process review and the staff retraining. Five of these were confirmed as positive and were sent for assistance, of which three underwent a change of internal channels due to the presence of micro-lesions. A total of 15 out of 152 endoscopes were quarantined because they were used on patients colonized/infected by alert microorganisms, but none gave a positive result at the next culture.

The outer surfaces of the endoscope valves were included in the microbiological surveillance, and high microbial counts (>100 CFU) were detected in only 6 out of 159 (3.7%) samples (mean load of 438 ± 87), while *E. coli* was isolated on two occasions, from the bioptic valve of a gastroscope and also in a colonoscope. After the introduction of hydrogen peroxide vapor sterilization of the valves, the positivity to the cultures was progressively eliminated. Moreover, to reduce the possibility of recontamination of the endoscope by its accessories, duodenoscopes were equipped with disposable valves when possible.

The water of the irrigation system was analyzed, with sampling inside the bottles, and 8 samples out of 12 showed microorganism growth. Considering that this water should be sterile, 67% of the samples were found to be non-compliant. 

The quality of the water used in the high-disinfection automatic reprocessor was found to be compliant with standards (20 samples), except for the inlet water, where the values of TVCs at 36 °C exceeded the legal limit in 4 out of 20 samples collected over the year (mean loads 170 ± 127 CFU/mL), demonstrating the effectiveness of the pre-filtration system installed upstream of the machines.

Finally, the analyses carried out to evaluate the hygienic quality of the storage cabinets, ventilated and not ventilated, were compliant (18/18).

## 4. Discussion

Routine microbiological surveillance of flexible endoscopes after reprocessing, during storage or before use is not recommended in U.S. standards [28,29], except for that performed on duodenoscopes [23,24]. The American Society for Microbiology has underlined the importance of microbiological analysis only for epidemiological investigation to verify the role of these devices in infection transmission or to evaluate the effectiveness of new or modified cleaning disinfection procedures [30]. Even the recent multidisciplinary guidelines of the American Society for Gastrointestinal Endoscopy and the Society for Healthcare Epidemiology of America do not support a requirement to systematically carry out microbiological surveillance [17,18].

Following cases of *KPC*-producing *Klebsiella pneumoniae* infection associated with ERCP [6], the Centers for Disease Control and Prevention issued a new guideline in 2015 [23] in which the importance of microbiological surveillance to ensure safety in the use of duodenoscopes was supported. This document was updated in 2018 [24].

Microbiological investigation is recommended in the guidelines of various international organizations, including the Gastroenterological Society of Australia and European organizations such as the European Society of Gastrointestinal Endoscopy and the European Society of Gastroenterology and Endoscopy Nurses and Associates. In Europe, there is variability in the suggested periodicity of such investigations: a quarterly frequency for analyses of endoscopes and washer disinfectors is proposed by German and Dutch guidelines, while in Austrian guidelines the proposed frequency is once a year and in Italian guidelines every six months.

The updated CDC guidelines identify an algorithm for the sampling of duodenoscopes and the process of verification, defining as a priority the sampling of duodenoscopes after 60 ERCP procedures or at least once a month, and every time that the device has been used on a patient for whom the status of colonization/infection with MDR microorganisms is known.

Microbiological surveillance should be carried out as a regular quality control for the prevention of infections associated with the use of these devices, but the frequency of microbiological investigations should be established after identifying the known or foreseeable hazards in the various phases of the process, an evaluation that must be carried out by the manager of the process (UNI TR16442). Environment, equipment and practice are considered to be significant risk factors for the transmission of infection, so healthcare organizations need to have effective mechanisms in place to control these risks.

In the ESGE–ESGENA Position Statement published in 2018, regular microbiological surveillance was recommended but no indication was given concerning the protocol to be used, referring to the previous guidelines for the area/material to be investigated and test methods to apply [20]. The sampling method recommended for routine tests was the flush method, involving the passage of liquid through the channels. This method can collect only the sessile cells of the biofilm and not the benthic ones, for which it is necessary to resort to the use of the brush, as suggested by the flush-brush-flush method mentioned in the CDC guideline [23]. Cori Ofstead et al. [31] verified that a greater proportion of positive samples could be obtained with this method compared with previous findings and Cattoir et al. demonstrated that the association of brushing with the endoscope sampling procedure increased the yield of the microbial surveillance culture [32].

Johani et al. reported that 77% of the microorganisms contaminating endoscopes after use are of environmental origin while 23% derive from the patient, both acting together to form a strong biofilm, tolerating decontamination procedures [33]. Furthermore, the longer an endoscope has been used, the more likely it is to be contaminated with biofilm [34]. The possible presence of biofilm therefore justifies the use of the flush-brush-flush method, which is considered the only suitable sampling method.

During the investigations conducted in the large Digestive Endoscopy Unit, an improvement in the quality of reprocessing was achieved through a careful analysis process and the carrying out of regular microbiological surveillance, adopting the flush-brush-flush method. This permitted the identification of endoscopes still contaminated, despite the execution of the correct reprocessing procedure, thus making it possible block their clinical use and reducing the risk of cross-contamination. This subsequently lowered the probability of endoscopes harboring high-concern organisms. According to CDC guidelines, endoscopes should not used until a negative microbiological report is issued; this means that the DEU requires a sufficient number of endoscopes to ensure that activity is not interrupted, whilst the laboratory must ensure the rapid release of the reports in order to guarantee a rapid turn-over of the instrumentation.

The corrective actions implemented following the results obtained during the first and the second phases of the surveillance led to a reduction in the contaminated endoscopes. The reduction was less marked in the second phase due to the adoption of the new sampling method. The flush-brush-flush method is known to have a greater capacity for detecting microbial contaminants. After five years of maintaining this implementation program, only 23% of endoscopes were found to be contaminated (35/152) and only five with microbiological non-conformity in the repeated samples (5/35, 14.3%). Three out of five endoscopes needed to have the internal channels replaced by the manufacturer due to the presence of micro-lesions; once back in use they did not show more positivity. A total of 15 out of 35 contaminated endoscopes were duodenoscopes (15 of 72 duodenoscopes, 20.8%). These results are in line with the findings of the cross-sectional study by Rauwers et al. [11], in which 22 of 155 duodenoscopes (22%) from 26 Dutch ERCP centers were found to be contaminated, mainly with enterobacteria, including *Enterobacter cloacae*, *Escherichia coli* and *Klebsiella pneumonia.*

These results were also obtained thanks to the improvement of the storage phase enabled by the use of dedicated ventilated cabinets, which were not previously present in the DEU. Accurate endoscope drying is crucial, whereas a humid environment facilitates microbial growth during storage. Studies have shown that appropriate storage can lead to a decrease in microbial contamination levels [35], although there is still considerable debate regarding the maximum length of time that reprocessed endoscopes can be stored without requiring re-disinfection. 

Water in the irrigation bottles, used during endoscopies for endoscope and lens washing, was recognized as a source of endoscope contamination. The finding of *P. aeruginosa* in these bottles may have been due to the ineffectiveness of the irrigation system anti-reflux valves when used with flexible gastrointestinal endoscopes, as underlined by the FDA [36]. When a single water bottle is used for multiple patients without reprocessing between patients or at the end of the day, backflow from irrigation channels into the water bottle can occur if the irrigation channel does not have a backflow-prevention mechanism in place. This problem was highlighted in the DEU and immediately solved using consumable 24-h multi-patient use devices with backflow valves.

Reusable valves for endoscope channels can be used multiple times and are intended to be reprocessed between patients; however, it is extremely difficult to visually confirm that these valves have been effectively cleaned, disinfected and thoroughly dried after each use. The use of disposable valves, in particular for the biopsy channel, mitigates this risk and enables a traceability that is not possible with reusable valves.

Although this risk assessment and management approach is costly in terms of human and economic resources—for example, in terms of the acquisition of equipment compliant with ISO standards—it is considered the most cost-effective approach for mitigating carbapenem-resistant enterobacteria (CRE) transmission risks during ERCP in settings with extremely high CRE prevalence [37].

In our opinion, this study is among a small number that demonstrate how to manage the risk associated with the reprocessing of digestive endoscopes—including the most crucial ones, such as duodenoscopes and echoendoscopes—through risk assessment at every stage of the process. In this context, microbiological surveillance is an important outcome indicator for the prevention of infections associated with the use of these devices. As reported in the last multisociety guideline on reprocessing flexible gastrointestinal endoscopes and accessories [38], despite their limitations, surveillance microbial cultures remain the most reliable indicators of residual contamination on reprocessed endoscopes.

## 5. Conclusions

The real infectious risk associated with the use of endoscopes is not yet known because not all the endoscopy centers have implemented regular microbiological surveillance of reprocessed endoscopes. Only through the close association between clinical strains and strains isolated from devices is it possible to define the origin of the infection. It is therefore desirable that microbiological surveillance be performed in centers where there is strong prevalence of high-concern organisms, at least for the endoscopes considered to be the most crucial (duodenoscopes and echoendoscopes) and in situations where delays in the reprocessing phases may presage the contamination of the endoscope and biofilm formation.

Microbiological surveillance is the starting point for the training of healthcare professionals in strict compliance with procedures recommended by the guidelines; the human factor can influence the outcome of the process and, consequently, the continuous training of operators is important to improve the perception of risk and reduce the monotony of the routine. Although it is advisable to have automatic equipment compliant with the standards, high costs should not preclude the implementation of other corrective actions that can improve the safety of the process.

The implementation of this process requires a significant effort by the endoscopy centers and the hospital medical directors, which must be evaluated in terms of cost effectiveness on the basis of the prevalence of these infections considering the possible use of completely disposable devices.

## Figures and Tables

**Table 1:** High-concern organisms identified during the first phase of microbiological surveillance.

Type of Endoscope (Number of Samples)	Microorganism	Swab from Biopsy Channel	Swab from Suction Channel	Swab from Air/Water Channel	Swab from Elevator Channel	Liquid from Internal Channels
		High-Concern Organisms				
**Duodenoscope** **(4)**	*E. coli*	2/4 (50%)	0	1/4 (25%)	1/4 (25%)	2/4 (50%)
	*K. pneumoniae*	0	0	0	1/4 (25%)	3/4 (75%) (2/3 *KPC*-*K. pneumoniae*)
	*P. aeruginosa*	1/4 (25%)	1/4 (25%)	0	0	1/4 (25%)
	***Total viable count*** *(quantity range and % NC samples)*	-	-	-	-	27–>300 CFU 3/4 (75%)
**Gastroscope** **(2)**	*E. coli*	0	0	0	0	1/2(50%)
	*K. pneumoniae*	0	0	0	0	0
	*P. aeruginosa*	0	0	0	0	0
	***Total viable count*** *(quantity range and % NC samples)*	-	-	-	-	220–>300 CFU 2/2 (100%)
**Colonoscope** **(4)**	*E. coli*	0	0	0	0	1/4 (25%)
	*K. pneumoniae*	0	0	0	0	0
	*P. aeruginosa*	0	1/4 (25%)	0	0	0
	***Total viable count*** *(quantity range and % NC samples)*	**-**	**-**	**-**	**-**	50–>300 CFU 1/4 (25%)

NC: non-compliant (TVC > 20 CFU/channel or >100 CFU/channels, according to ESGE/ESGENA or CDC guidelines, respectively).

**Table 2:** High-concern organisms identified during the second phase of microbiological surveillance.

Type of Endoscope (Number of Samples)	Microorganism	Swab from Biopsy Channel	Swab from Suction Channel	Swab from Air/Water Channel	Swab from Elevator Channel	Liquid from Internal Channels
		High-Concern Organisms				
**Duodenoscope** **(10)**	*S. maltophilia*	0	0	0	0	1/10 (10%)
	*K. pneumoniae* *K. oxytoca*	0	0	0	0	4/10 (40%) (1/4 *KPC-K. oxytoca*)
	***Total viable Count*** *(quantity range and % NC samples)*	-	-	-	-	50–>300 CFU 2/10 (20%)
**Gastroscope** **(1)**	*P. fluorescens*	0	0	0	0	1/1 (100%)
	***Total viable count*** *(quantity range and % NC samples)*	-	-	-	-	>300 CFU 1/1 (100%)
**Colonoscope** **(6)**	*P. aeruginosa* *P. fluorescens*	0	0	2/6(33.3%)	0	3/6(50%)
	***Total viable count*** *(quantity range and % NC samples)*	**-**	**-**	**-**	**-**	20–>300 CFU 4/6 (66%)

NC: non-compliant (TVC > 20 CFU/channel or >100 CFU/channels, according to ESGE/ESGENA or CDC guidelines, respectively).

**Table 3:** High-concern organisms isolated during the third phase of microbiological surveillance.

Type of Endoscope (Number of Samples)	Microorganism	Swab from Biopsy Channel	Swab from Water Channel	Swab from Air Channel	Swab from Elevator Channel	Liquid from Internal Channels
		High-Concern Organisms				
**Duodenoscope** **(72)**	*K. pneumoniae*	0	0	0	0	3/72 (4.2%)
	*S. epidermidis* *Staphylococcus* spp.	0	0	0	0	2/72 (2.8%)
	*E. cloacae*	0	0	0	0	1/72 (1.3%)
	*E. coli*	0	0	0	0	2/72 (1.3%)
	*P. aeruginosa* *P. stutzeri* *P. putida*	0	0	0	0	5/72 (4.2%)
	*A. baumannii* *A. lwoffii*	0	0	0	0	2/72 (2.8%)
	***Total viable Count*** *(quantity range and % NC samples)*	-	-	-	-	1–300 CFU 4/72 (5.5%)
**Gastroscope** **(32)**	*K. pneumoniae*	0	0	0	0	2/32 (6.2%) (NDM*-K. pneumoniae*)
	*E. cloacae*	0	0	0	0	1/32 (3.1%)
	*P.* *aeruginosa* *P. stutzeri* *P. mosselii* *P. fluorescens*	0	0	0	0	3/32 (9.4%)
	*S. maltophilia*	0	0	0	0	1/32 (3.1%)
	*A. lwoffii*	0	0	0	0	1/32 (3.1%)
	***Total viable count*** *(quantity range and % NC samples)*	-	-	-	-	1–83 CFU 4/32 (12.5%)
**Colonoscope** **(24)**	*E. coli*	0	0	0	0	3/24 (12.5%)
	*Staphylococcus* spp.	0	0	0	0	2/24 (8.3%)
	*P. aeruginosa*	0	0	0	0	2/24 (8.3%)
	***Total viable count*** *(quantity range and % NC samples)*	**-**	**-**	**-**	**-**	1–>300 CFU 4/24 (16.7%)

NC: non-compliant (TVC > 20 CFU/channel or >100 CFU/channels, according to ESGE/ESGENA or CDC guidelines, respectively).

## Data Availability

The data were collected and processed directly by the authors so we have no link to suggest.
